# Accuracy of Augmented Reality–Assisted Navigation in Dental Implant Surgery: Systematic Review and Meta-analysis

**DOI:** 10.2196/42040

**Published:** 2023-01-04

**Authors:** Hang-Nga Mai, Van Viet Dam, Du-Hyeong Lee

**Affiliations:** 1 Institute for Translational Research in Dentistry Kyungpook National University Daegu Republic of Korea; 2 Hanoi University of Business and Technology Hanoi Vietnam; 3 Department of Implantology Hanoi National Hospital of Odonto-stomatology Hanoi Vietnam; 4 VNU School of Medicine and Pharmacy Vietnam National University Hanoi Vietnam; 5 Department of Prosthodontics School of Dentistry Kyungpook National University Daegu Republic of Korea

**Keywords:** augmented reality, accuracy, computer-guided surgery, dental implants, systematic review, meta-analysis

## Abstract

**Background:**

The novel concept of immersive 3D augmented reality (AR) surgical navigation has recently been introduced in the medical field. This method allows surgeons to directly focus on the surgical objective without having to look at a separate monitor. In the dental field, the recently developed AR-assisted dental implant navigation system (AR navigation), which uses innovative image technology to directly visualize and track a presurgical plan over an actual surgical site, has attracted great interest.

**Objective:**

This study is the first systematic review and meta-analysis study that aimed to assess the accuracy of dental implants placed by AR navigation and compare it with that of the widely used implant placement methods, including the freehand method (FH), template-based static guidance (TG), and conventional navigation (CN).

**Methods:**

Individual search strategies were used in PubMed (MEDLINE), Scopus, ScienceDirect, Cochrane Library, and Google Scholar to search for articles published until March 21, 2022. This study was performed in accordance with the PRISMA (Preferred Reporting Items for Systematic Reviews and Meta-Analyses) guidelines and registered in the International Prospective Register of Systematic Reviews (PROSPERO) database. Peer-reviewed journal articles evaluating the positional deviations of dental implants placed using AR-assisted implant navigation systems were included. Cohen *d* statistical power analysis was used to investigate the effect size estimate and CIs of standardized mean differences (SMDs) between data sets.

**Results:**

Among the 425 articles retrieved, 15 articles were considered eligible for narrative review, 8 articles were considered for single-arm meta-analysis, and 4 were included in a 2-arm meta-analysis. The mean lateral, global, depth, and angular deviations of the dental implant placed using AR navigation were 0.90 (95% CI 0.78-1.02) mm, 1.18 (95% CI 0.95-1.41) mm, 0.78 (95% CI 0.48-1.08) mm, and 3.96° (95% CI 3.45°-4.48°), respectively. The accuracy of AR navigation was significantly higher than that of the FH method (SMD=−1.01; 95% CI −1.47 to −0.55; *P*<.001) and CN method (SMD=−0.46; 95% CI −0.64 to −0.29; *P*<.001). However, the accuracies of the AR navigation and TG methods were similar (SMD=0.06; 95% CI −0.62 to 0.74; *P*=.73).

**Conclusions:**

The positional deviations of AR-navigated implant placements were within the safety zone, suggesting clinically acceptable accuracy of the AR navigation method. Moreover, the accuracy of AR implant navigation was comparable with that of the highly recommended dental implant–guided surgery method, TG, and superior to that of the conventional FH and CN methods. This review highlights the possibility of using AR navigation as an effective and accurate immersive surgical guide for dental implant placement.

## Introduction

The clinical success of dental implant placement requires proper preoperative implant planning along with an effective and accurate surgical process [1,2]. Computer-assisted implant surgery (CAIS), also referred to as guided dental implant surgery, is a more effective and accurate method than the conventional freehand (FH) method [3-6]. On the basis of the surgical guidance phase, CAIS can be classified into static and dynamic systems [7]. Although both systems share a similar presurgical planning phase wherein the dental implant positioning process is simulated based on cone-beam computed tomography (CBCT) and intraoral scan images of patients using dedicated dental implant planning software programs [8,9], they differ in the surgical phase. In the surgical phases of static CAIS, implant-guided surgery can be performed using a static guide template that transfers the planned position of the dental implant to the surgical site by guiding sleeves or keys, depending on the specific guiding system [1,8,9]. In contrast to the static approach, dynamic CAIS uses a dynamic navigating system that allows real-time tracking of the surgical instrument relative to the patient and dynamically displays the position of the drill on CBCT during dental implant placement [8-10].

Static dental implant–guided surgery has been widely used in clinical practice, because it results in highly accurate implant placement [4]. However, the inability to change the presurgical plan is considered the main limitation of static dental implant–guided surgery [11]. In addition, there are several drawbacks associated with the use of a rigid surgical template, such as an obstructed view of the surgical site and an increase in the heat generated during osteotomy owing to the reduced contact of cooling fluids with the drills [2,12-14]. Surgical templates can be problematic for patients with limited mouth opening or those requiring simultaneous bone grafting [15,16]. Moreover, surgical templates largely rely on support tissues; thus, the accuracy of dental implant placement is significantly reduced in cases of unstable or poor support conditions [17]. Dynamic navigation was introduced to overcome the limitations of static guiding systems.

Current dynamic dental implant–guiding systems often consist of 2 main components: an optical tracking device that records either passive or active tracking arrays positioned on patients and surgical instruments and a navigation system that displays the positions of the drill on CBCT images [8,18]. The advantages of the dynamic approach include the real-time localization of surgical drills concerning critical anatomical structures, dynamic navigation of the drill along with a presurgical plan without the need for a rigid template, and providing operators with continuous real-time feedback during surgery [9,10]. Moreover, the accuracy of dynamic CAIS is clinically acceptable; hence, dynamic CAIS can be considered a promising method for precise dental implant placement [10,19]. Conventionally, dynamic CAIS displays tracking images on a separate screen monitor. Therefore, operators control the instruments while looking away from the oral cavity to follow the navigation [10]. This limitation not only interrupts the surgical flow and involves the risk of operation errors but also causes anxiety in operators during surgery [20].

Recently, augmented reality (AR)—an innovative image technology that produces an immersive surgical context by merging digital data with the real environment—has been incorporated into a dynamic dental implant–guiding system to help operators directly visualize digital navigation information over the actual operating field of view to improve real-time surgical performance [21-23]. AR technology has been increasingly used in the medical and dental fields owing to the rapid developments in image recognition and tracking technology [24-30]. Several AR-assisted navigation systems with various characteristics have been introduced, and their accuracy has attracted significant interest. However, compared with static and conventional dynamic navigation systems, AR-assisted navigation systems have been less frequently documented in the literature. To the best of our knowledge, no systematic review and meta-analysis study has been published regarding the accuracy of AR-assisted dynamic dental implant-guiding surgery.

Therefore, we investigated the accuracy of innovative imaging technology in the form of an AR-assisted navigation system that can merge digital images in a real environment to help oral surgeons visualize virtually planned dental implant positions at actual surgical sites. The primary aim of this study was to evaluate the accuracy of the recently developed AR-assisted dental implant navigation system (AR navigation) in terms of positional deviations between the planned and placed implants. The secondary aim of this study was to compare the accuracy of AR navigation with that of other dental implant placement methods, including the FH method, template-based static guidance (TG) method, and conventional navigation (CN) method.

## Methods

### Study Protocol

This study was performed in accordance with the PRISMA (Preferred Reporting Items for Systematic Reviews and Meta-Analyses) guidelines [31] and registered in the International Prospective Register of Systematic Reviews (PROSPERO) database (registration no. CRD42022323650). The study was designed based on the population, intervention, comparison, and outcomes question as follows:

1. Population (P): Dental implants, implant pins, implant drilling channels placed on humans or animals or simulated clinical models
2. Intervention (I): Implant surgery guided by AR navigation (AR navigation group)
3. Comparison (C): Implant surgery guided by other methods such as the FH method (FH group), TG method (TG group), and CN method (CN group)
4. Outcomes (O): Positional deviations of the placed implant or drilling channel described by the differences between the planned and actual positions of the placed implants
5. Study (S): Randomized and nonrandomized, observational (analytic and descriptive), and preclinical or in vitro studies

### Searching Strategy

The following electronic databases were searched: PubMed, Scopus, Web of Science, Cochrane Library, and Google Scholar. In addition, the references cited in the full-text and relevant articles in several dental journals such as *Journal of Clinical Periodontology*, *Periodontology 2000*, *Journal of Periodontology*, *Clinical Oral Implant Research*, and *Clinical Implant Dentistry and Related Research* were manually searched. The search strategy for each database was established using Boolean logic (AND, OR, NOT) to combine search terms. The individual Boolean operators established for searching for each data source are listed in Table 1. The retrieved articles were sorted using reference manager software (EndNote; version x9.2; Clarivate Analytics Inc).

**Table 1:** Boolean operators for automatic searching of each data source (Total N=425).

Database	Boolean operator	Result, n
PubMed	(Implant Surgical Procedure*[Title/Abstract] OR Implant surg*[Title/Abstract] OR dental implant surg*[Title/Abstract] OR oral implant surg*[Title/Abstract] OR dental implant*[Title/Abstract] OR dental implant prosthe*[Title/Abstract] OR intraoperative implant surg*[Title/Abstract] OR maxillofacial implant surg*[Title/Abstract] OR craniofacial implant surg*[Title/Abstract] OR navigated implant surg*[Title/Abstract] OR guided implant surg*[Title/Abstract] OR implantoplasty*[Title/Abstract]) AND (augmented reality[Title/Abstract] OR hybrid reality[Title/Abstract] OR mixed reality[Title/Abstract] OR AR[Title/Abstract] OR head mounted display[Title/Abstract] OR heads up display*[Title/Abstract] OR MR[Title/Abstract] OR immersive technologies[Title/Abstract]) AND (accuracy*[Title/Abstract] OR trueness*[Title/Abstract] OR precision*[Title/Abstract] OR reproducibility*[Title/Abstract] OR reliability*[Title/Abstract] OR dimensional accuracy[Title/Abstract] OR comparison[Title/Abstract] OR evaluation*[Title/Abstract] OR verify*[Title/Abstract] OR valid*[Title/Abstract])	43
Scopus	TITLE-ABS-KEY (“implant surgery” OR “prosthetic dental Implant” OR “implant placement” OR “oral implant surgery” OR “dental implant” OR “dental implant prosthesis” OR “intraoperative implant surgery” OR “maxillofacial implant surgery” OR “craniofacial implant surgery” OR “navigated implant surgery” OR “guided implant surgery” OR “implant navigation” OR “virtual implant surgery planning” OR “dynamic implant surgery planning” OR “implantoplasty”) AND TITLE-ABS-KEY (“augmented reality” OR “hybrid reality” OR “mixed reality” OR “AR” OR “head-mounted display” OR “heads up display” OR “MR” OR “immersive technologies”) AND TITLE-ABS-KEY (“accuracy” OR “precision” OR “trueness” OR “reliability” OR “dimensional accuracy” OR “evaluation” OR “verify” OR “validation”)	64
Cochrane Library	(“implant surgery” OR “prosthetic dental Implant” OR “implant placement” OR “oral implant surgery” OR “dental implant” OR “dental implant prosthesis” OR “intraoperative implant surgery” OR “maxillofacial implant surgery” OR “craniofacial implant surgery” OR “navigated implant surgery” OR “guided implant surgery” OR “implant navigation” OR “virtual implant surgery planning” OR “dynamic implant surgery planning” OR “implantoplasty”):ti,ab,kw AND (“augmented reality” OR “hybrid reality” OR “mixed reality” OR “AR” OR “head-mounted display” OR “heads up display” OR “MR” OR “immersive technologies”):ti,ab,kw AND (“accuracy” OR “precision” OR “trueness” OR “reliability” OR “dimensional accuracy” OR “evaluation” OR “verify” OR “validation”):ti,ab,kw	15
Embase	(“implant surgery”:ti,ab w OR “prosthetic dental implant”:ti,ab,kw OR “implant placement”:ti,ab,kw OR “oral implant surgery”:ti,ab,kw OR “dental implant”:ti,ab,kw OR “dental implant prosthesis”:ti,ab,kw OR “intraoperative implant surgery”:ti,ab,kw OR “maxillofacial implant surgery”:ti,ab,kw OR “craniofacial implant surgery”:ti,ab,kw OR “navigated implant surgery”:ti,ab,kw OR “guided implant surgery”:ti,ab,kw OR “implant navigation”:ti,ab,kw OR “virtual implant surgery planning”:ti,ab,kw OR “dynamic implant surgery planning”:ti,ab,kw OR “implantoplasty”:ti,ab,kw) AND (“augmented reality”:ti,ab,kw OR “hybrid reality”:ti,ab,kw OR “mixed reality”:ti,ab,kw OR “ar”:ti,ab,kw OR “head mounted display”:ti,ab,kw OR “heads up display”:ti,ab,kw OR “mr”:ti,ab,kw OR “immersive technologies”:ti,ab,kw) AND (“accuracy”:ti,ab,kw OR “precision”:ti,ab,kw OR “trueness”:ti,ab,kw OR “reliability”:ti,ab,kw OR “dimensional accuracy”:ti,ab,kw OR “evaluation”:ti,ab,kw OR “verify”:ti,ab,kw OR “validation”:ti,ab,kw)	33
Web of science	([TS=(“implant surgery” OR “prosthetic dental Implant” OR “implant placement” OR “oral implant surgery” OR “dental implant” OR “dental implant prosthesis” OR “intraoperative implant surgery” OR “maxillofacial implant surgery” OR “craniofacial implant surgery” OR “navigated implant surgery” OR “guided implant surgery” OR “implant navigation” OR “virtual implant surgery planning” OR “dynamic implant surgery planning” OR “implantoplasty”)] AND TS=[“augmented reality” OR “hybrid reality” OR “mixed reality” OR “AR” OR “head-mounted display” OR “heads up display” OR “MR” OR “immersive technologies”]) AND TS=(“accuracy” OR “precision” OR “trueness” OR “reliability” OR “dimensional accuracy” OR “evaluation” OR “verify” OR “validation”)	41
Google Scholar	“dental implant surgery”~ AND “augmented reality”~ AND “accuracy”~	219
References from related articles	Manual search	6
Dental journals	Manual search	4

### Inclusion and Exclusion Criteria

This systematic review considered all original studies published until March 21, 2022 (ie, clinical trials; cohort studies; case-control studies; and preclinical, in vitro, and ex vivo studies) that evaluated the accuracy of AR navigation without any language restrictions. Studies without metric measurements for reporting implant positions and those that were unrelated to dental implant placement were excluded. Similarly, systematic or narrative reviews without implant placement accuracy assessments, letters to the editor, author or editorial opinion articles, and epidemiological studies were excluded. Gray literature and unpublished data were not included in this study to avoid a possible research bias. Only studies that reported deviation values between planned and executed implant positions were included in the meta-analysis. The detailed inclusion and exclusion criteria are listed in Table 2.

**Table 2:** Inclusion and exclusion criteria for this study.

	Inclusion criteria	Exclusion criteria
Study type	Randomized or nonrandomized controlled clinical trials; cohort studies; case-control studies; and preclinical, in vitro, and ex vivo studies	Letters to the editor, author or editorial opinion articles, epidemiological studies, gray literature, and unpublished data
Study subject	Dental implants, implant pins, implant drilling channels placed on humans or animals, and clinical simulated models	Implants that are not for dental use
Interventions	Implant surgery guided by AR^a^ implant navigation, freehand method, template-based static guidance, and conventional navigation	Studies without implant placement guided by AR implant navigation
Measurements for evaluating the accuracy of the implant placement method	Positional deviations between the planned and actual placed implants that are expressed in numeric measurements	Studies without metric measurements
Outcomes	Lateral coronal deviation (mm), lateral apical deviation (mm), global coronal deviation (mm), global apical deviation (mm), depth deviation (mm), and angular deviation (°)	Studies where the positions of the placed implants could not be determined

^a^AR: augmented reality.

### Study Selection and Data Extraction

For study selection, 2 reviewers (HNM and DHL) independently screened and selected eligible studies based on inclusion and exclusion criteria. The agreement between reviewers was calculated using Cohen κ coefficient. Any disagreement was resolved through a discussion between the reviewers or consultation with an expert (VVD). Relevant information was extracted from the eligible studies, which included study characteristics (ie, authors, year of publication, and study design), participant characteristics (ie, number of participants, type of jaws, number of jaws, type of edentulism, position of the implant in the jaws, and number of implants or drilling channels), AR system characteristics (ie, type of digital image projected on the surgical site, tracking technology, user interface, and image display technology), the outcome of interest (ie, deviation of image-to-registration and positional deviations between planned and placed implants), comparison groups (ie, FH method, CN method, TG method, and traditional dynamic guiding system), and major findings from each included study. In case of missing or ambiguous data, the corresponding authors were contacted for data clarification. Information was extracted independently by 2 reviewers and recorded on an electronic spreadsheet (Microsoft Office Excel 2019; Microsoft Corp). After the initial data extraction, the final data table was re-evaluated by both reviewers, and inconsistencies were corrected either by discussion between the 2 reviewers or by consultation with a third reviewer (VVD).

### Risk of Bias and Publication Bias Assessment

The risk of bias in the included studies was assessed using the Risk Of Bias In Non-randomized Studies of Interventions tool [32] based on seven domains: (1) bias owing to confounding, (2) bias in the selection of study participants, (3) bias in the classification of interventions, (4) bias owing to deviations from intended interventions, (5) bias owing to missing data, (6) bias in outcome measurement, and (7) bias in the selection of the reported result. The overall risk of bias in the included studies was judged as “low risk” if all the domains were judged to have a low risk of bias, “moderate risk” if all or some domains were judged to have a moderate risk of bias, as “serious risk” if at least one domain was judged to have a serious risk of bias, and as “critical risk” if at least one domain was judged to have a critical risk of bias. The “no information” category was used for studies in which insufficient data were reported to permit judgment. Publication bias in the included studies was assessed using Egger linear regression statistical test [33]. The Duval and Tweedie trim-and-fill method [34] was used to further assess the effects of publication bias and redress asymmetry.

All statistical analyses were performed using R statistical software (version 3.6.0; R Foundation for Statistical Computing), and the significance level was set at .05. Weighted bars and traffic light plots were used to visualize the results of the risk of bias assessments, and funnel plots were generated to visually report publication bias.

### Meta-analysis

**Figure 1:**
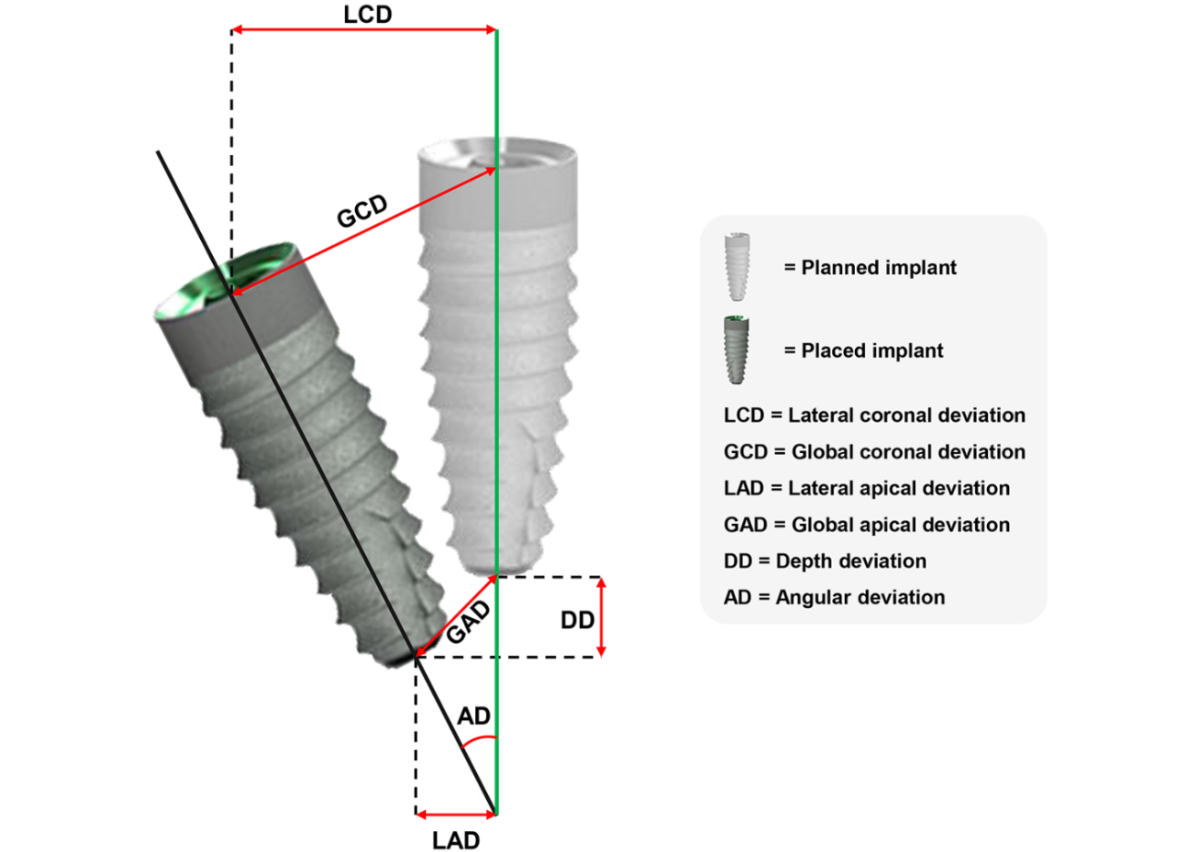
Positional deviations between the planned and placed implants.

The positional deviations between the planned and placed implants were analyzed using 6 outcome variables: lateral coronal deviation (LCD), lateral apical deviation (LAD), global coronal deviation (GCD), global apical deviation (GAD), depth deviation, and angular deviation (Figure 1).

To obtain a general view of the accuracy of implant placement performed using AR navigation, single-arm meta-analyses for continuous data that involved estimating the mean deviations for each deviation variable were used for data analysis. Weighted means with 95% CIs were assessed, and subgroup analyses were performed according to deviation outcome variables (ie, lateral, global, depth, and angular deviations).

To compare the accuracy of AR navigation (experimental group) with other implant placement methods (control groups), 2-arm meta-analyses for continuous data were performed. The standardized mean difference (SMD) with a 95% CI between the experimental and reference data sets was analyzed to estimate the effect size [35]:



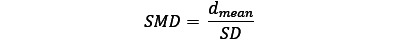



where d_mean_ is the difference in mean values between the experimental and control groups and SD of the measurements. Subgroup analyses were performed to compare the AR navigation group with the FH, TG, and CN navigation groups.

Meta-analyses were performed using either a random effects model or a fixed-effects model based on the heterogeneity among studies evaluated by the Higgins *I^2^* statistic [36]:



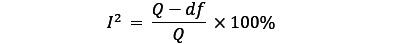



where *Q* is the chi-square statistic and *df* is the degree of freedom. A higher *I^2^* value indicates stronger heterogeneity across studies. In cases of *I*^2^≥50%, the data were considered statistically heterogeneous, and a random-effects model was used for data analysis.

## Results

### Study Selection

**Figure 2:**
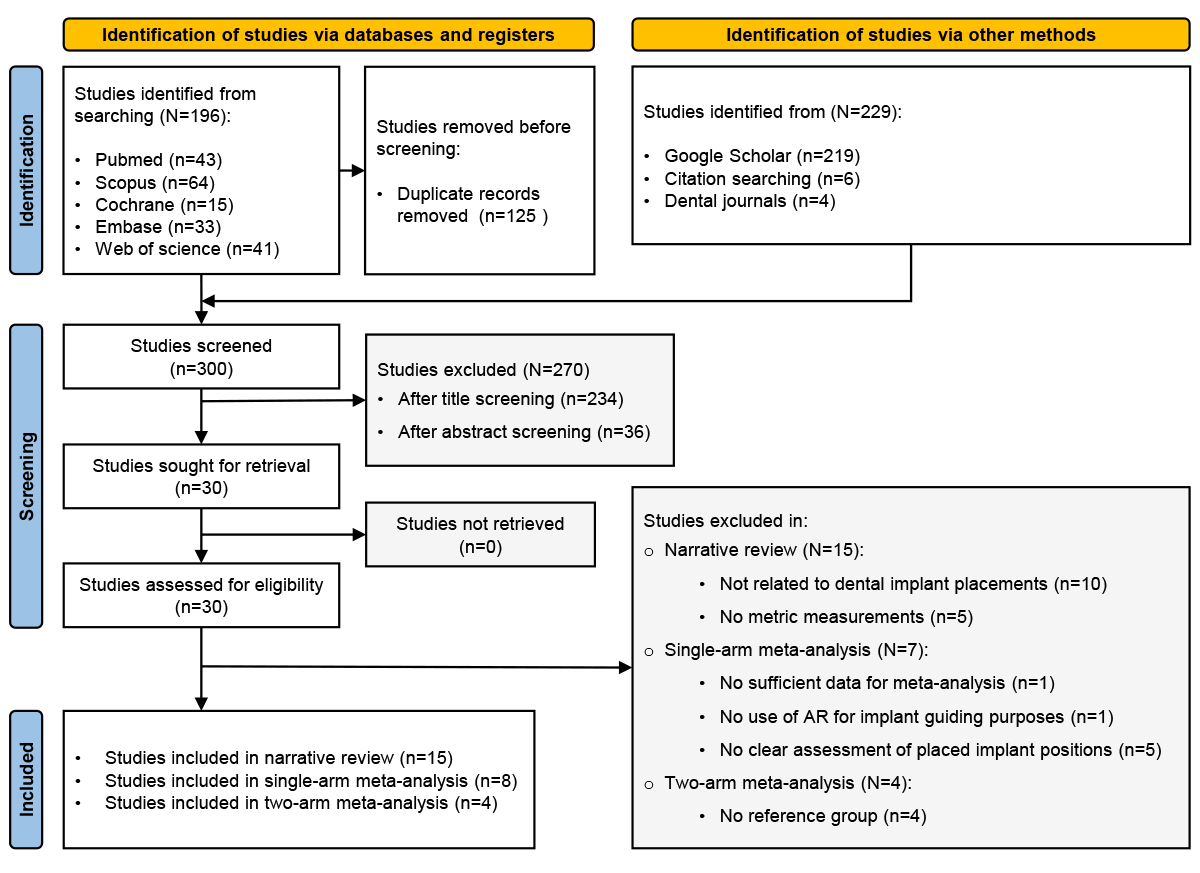
PRISMA (Preferred Reporting Items for Systematic Reviews and Meta-Analyses) flowchart showing the results of the study selection process.

The search results are shown in the PRISMA flowchart (Figure 2). In summary, 425 articles were selected after the initial search. The remaining 300 records were screened after excluding 125 duplicate articles. After screening the titles and abstracts, 270 irrelevant articles were excluded. Therefore, 30 articles were subjected to full-text assessment. Among them, 15 articles were considered eligible for narrative review, 8 articles were considered suitable for single-arm meta-analysis, and 4 articles were included in the 2-arm meta-analysis. Cohen κ coefficient showed a high degree of agreement between the 2 reviewers when selecting the studies (κ=0.88).

### Study Characteristics

The characteristics of the included studies are summarized in Multimedia Appendix 1 [20-23,37-47]. Among the 15 eligible studies, 5 studies [22,23,37,38] involved volunteers or patients, 2 studies were performed on cadavers [39-41], 5 studies [20,42-45] involved plastic models, and 3 studies [21,46,47] used actual patient casts. Regarding the type of edentulous jaw, the number of mandibles (98/157, 62.4%) was higher than that of maxillary jaws (59/157, 37.6%), and the amount of partial edentulism (9/14, 64%) was higher than that of complete edentulism (5/14, 36%). The number and type of edentulism were not reported in a previous study [23]. Regarding implant placements, 7 studies involved [22,37-41,45] dental implants, 2 studies [21,47] used pins, and 4 studies [42-44,46] exploited drill channels to measure implant positional deviations. Two studies [20,23] did not clarify the number of implant placements.

**Figure 3:**
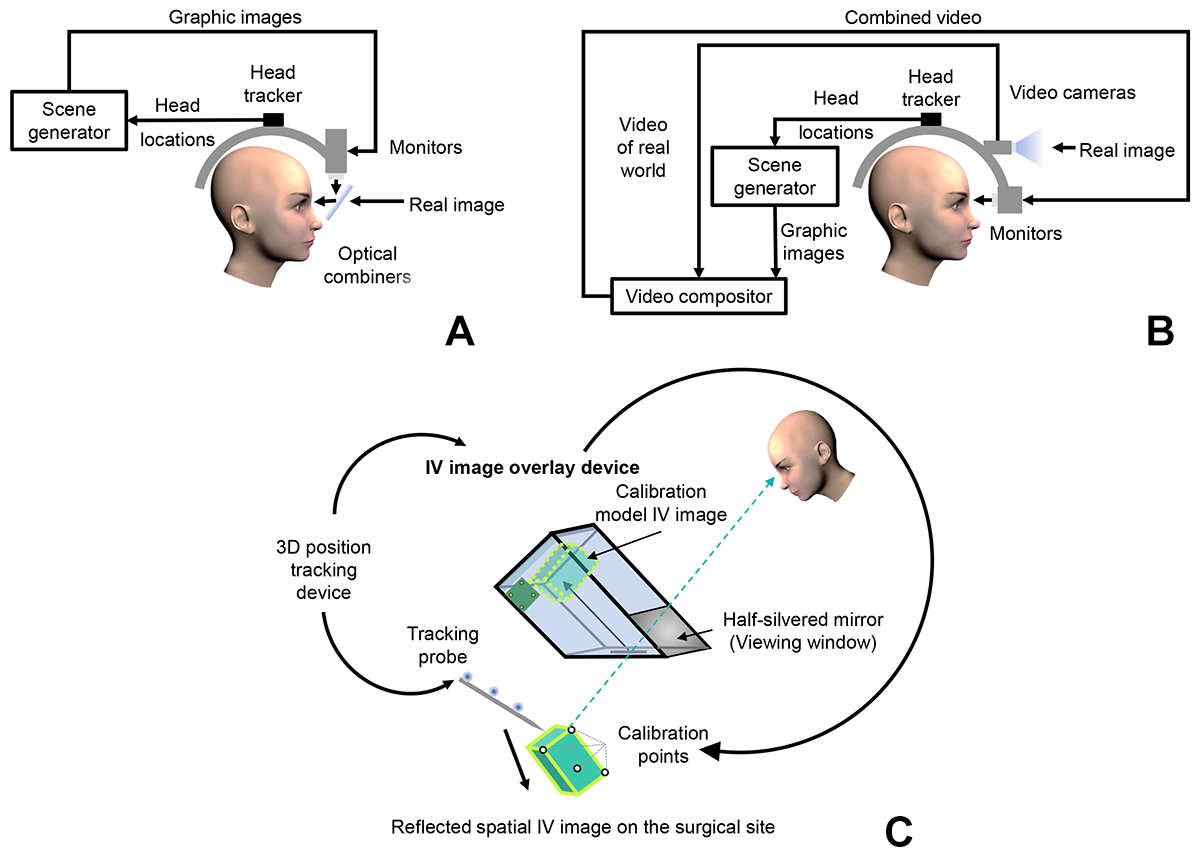
Augmented reality image display devices. (A) OST HMD device. (B) VST HMD. (C) IV image overlay device. HMD: head-mounted display; IV: integral videography; OST: optical see-through; VST: video see-through.

**Figure 4:**
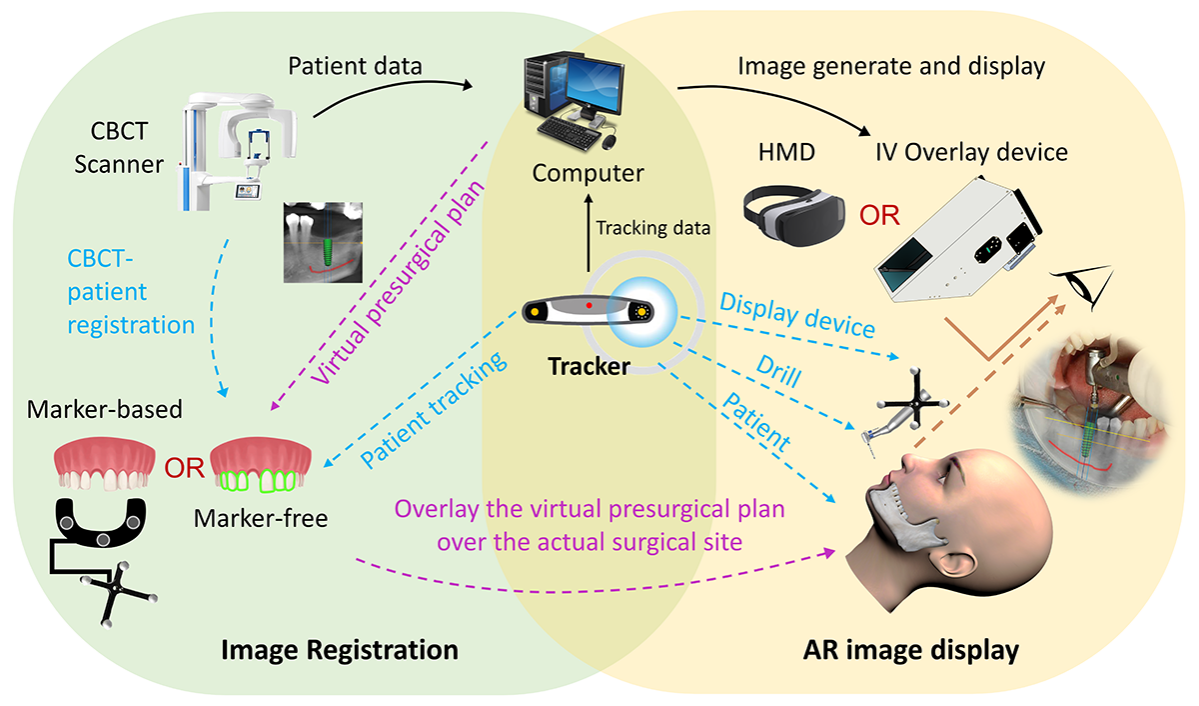
Augmented reality (AR) dental implant navigation system. CBCT: cone-beam computed tomography; HMD: head-mounted display device; IV: integral videography.

Regarding AR technologies, most AR-assisted implant navigation systems allow the appearance of virtually planned implants. Some systems provide additional guidance such as the surgical path or drilling axis of the drills and the alveolar nerve or mandibular canal. Some systems included a warning function to alert surgeons and aid adjustments to the surgical strategy. In particular, one study [46] adopted the AR technology to facilitate the use of static implant-guiding systems. Most studies applied marker-based tracking technology; only 3 studies [23,38,47] used marker-free tracking technology for real-time image displays during implantation. To display the AR image, a head-mounted display (HMD) was used in 7 studies [22,39,40,42,44-46], an integral videography (IV) overlay device was used in 5 studies [21,23,38,43,47], and a smartphone was used in 1 study [41]. The general structure of the AR image display devices is summarized in Figure 3, and detailed information on AR-assisted implant navigation systems reported in the included studies is provided in Figure 4 and Table 3. Among the studies that included a control group, 2 [21,45] included the FH group, 2 [41,45] included the TG group, and 4 [40,41,44,47] included the CN group as the control group.

**Table 3:** Augmented reality–assisted implant navigation systems reported in the included study.

Study	AR^a^ image display device	Optical tracking device	Computer and software
Wanschitz et al [39]	Varioscope AR is developing a system based on the Varioscope AF3 (Life Optics)	Optical tracking system (flash point 5000; Image Guided Technologies Inc)	The control computer for the HMD^b^ is a commercial PC with an Intel Pentium III 933-MHz processor. The displays of the Varioscope AR are driven by an ASUS 7100 GeForce 2 dual-head graphics board; the control software was developed using GCC 2.71 (Free Software Foundation) under SuSE Linux 7.1 (SuSE GmbH)
Yamaguchi et al [42]	Data glass 2 (SHIMAZU Inc)	Micron Tracker2 Sx60 (Claron Technology Inc)	ThinkPad X32 (IBM Inc)
Tran et al [43]	A half-silvered mirror is attached to the IV^c^ display device (NR^d^)	Optical tracking device (NR)	CPU: Pentium core 2 Duo, 2.4 GHz GPU: NVIDIA Quadro Plex model 4 (Quadro FX 5600 × 2); elemental IV image size: 1024 × 768 pixels
Yamaguchi [20]	Retinal Imaging Display (Prototype; Brother Industries, Ltd)	Optical tracker (Micron tracker 2 Sx60; Claron Technology Inc)	A laptop computer (Thinkpad X32, IBM) and a least median square method and an algorithm developed by the authors.
Vigh et al [44]	HMD nVisor SX60 (NVIS)	Polaris (Northern Digital Inc)	NR
Katić et al [40]	HMD (NR)	Polaris (Northern Digital Inc)	NR
Lin et al [46]	1280 × 720 resolution HMD (Sony HMZ-T1 personal 3D viewer)	Not required	A laptop computer with an Intel Core i7-2760QM 2.4 GHz (Intel Company).
Jiang et al [47]	Not required	Polaris (Northern Digital Inc)	The software developed by the Visualization Toolkit
Matsuo et al [37]	A head-mounted image processor unit (HMS-3000MT; Sony Corp)	NR	NR
Ma et al [21]	An IV overlay device includes a 9.7-in 3D display with a resolution of 1536 × 2048 pixels, which is covered by a microlens array with a lens pitch of 1.016 mm and a focal length of 3.0 mm (NR)	Polaris (Northern Digital Inc)	The computer was equipped with a high-performance GPU (GTX950, Nvidia Corp)
Pellegrino et al [22]	Virtual reality glasses (HoloLens, Microsoft)	Navigation system ImplaNav (BresMedical)	A dedicated software created by Fifthingenium
Wang et al [38]	Not required	A customized stereo camera is built to track the patient’s teeth (shape tracking; NR)	Digital scenes can be rendered and mixed with the camera’s stereo views using OpenGL APIs for AR surgical navigation (NR)
Shrestha et al [23]	NR	NR	The proposed system is implemented by using MATLAB R2019a; for the experiment, a system configured with Intel Core i5-3337U CPU@2.4 GHz and 4.00-GB RAM was used
Kivovics et al [45]	Head-mounted virtual retinal display (Magic Leap One, Magic Leap Inc)	Not required	Innooral (Innooral System, Innoimplant Ltd), MagicLeap The Lab (Magic Leap One, Magic Leap Inc), Net 4.6 (Microsoft Corp), and Microsoft Windows 10 (Microsoft Corp) operating system
Ochandiano et al [41]	IPhone 6 (Apple Inc) with a customized AR smartphone app (NR)	Polaris (Northern Digital Inc)	A customized AR smartphone apps was developed to project the patients’ digital models onto the real-world image (NR)

^a^AR: augmented reality.

^b^HMD: head-mounted display.

^c^IV: integral videography.

^d^NR: not reported.

### Risk of Bias Assessment

**Figure 5:**
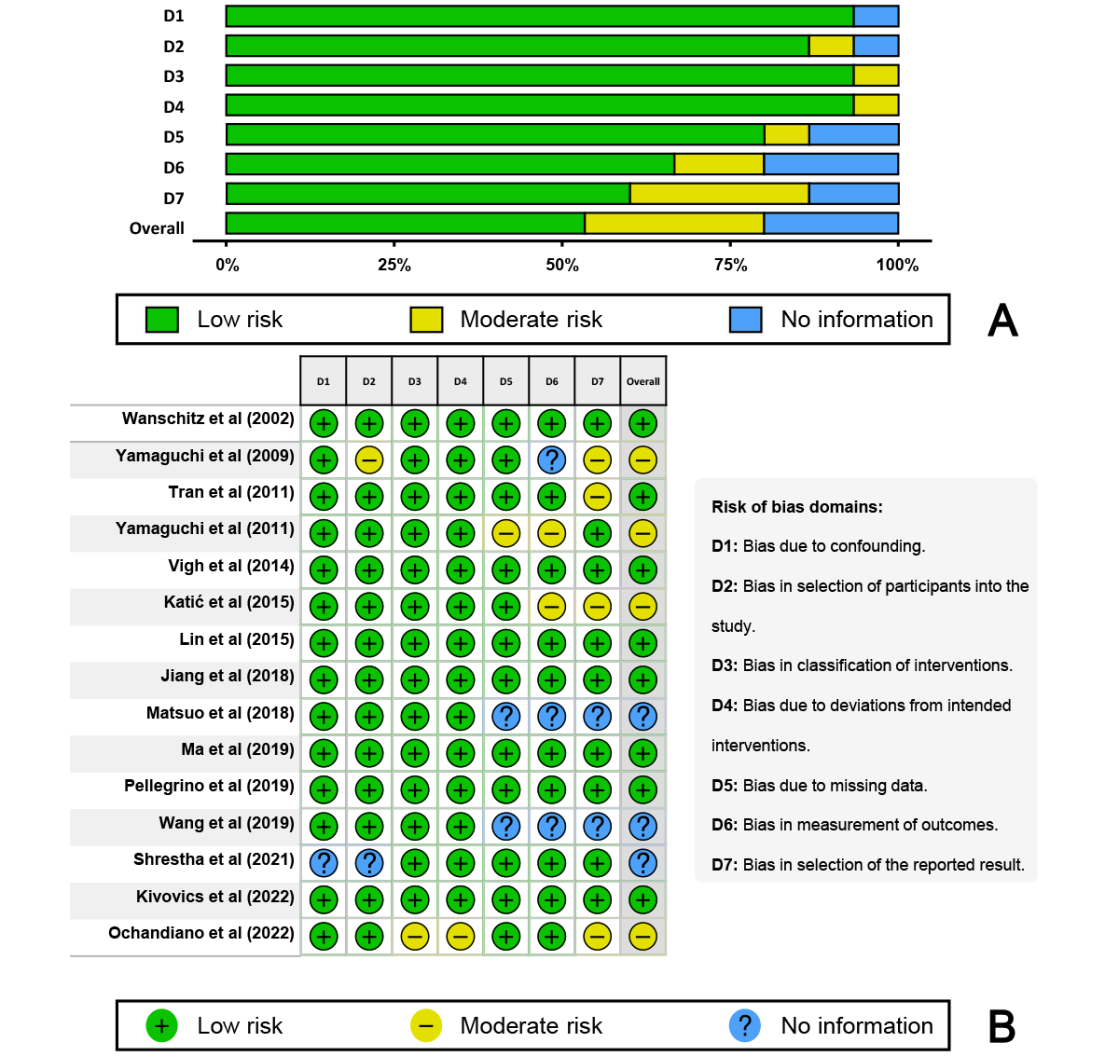
Risk of bias assessment using the Risk Of Bias In Non-randomized Studies of Interventions (ROBINS-I) tool. (A) Weighted bar plots showing the distribution of risk-of-bias judgments within each bias domain, and (B) traffic light plots of the domain-level judgments for each study.

The risk of bias assessment is shown in Figure 5. An overall moderate risk of bias was found in 4 studies [20,40-42]. Three studies [23,37,38] reported insufficient data to permit judgment. The main limitation of the included studies was the limited sample size, which might have affected the results, and some articles did not specify the blinding of the outcome assessment. Therefore, though there was no serious and critical risk of bias among the assessed domains, domain 6 (ie, bias in outcome measurement) and domain 7 (bias in the selection of the reported result) exhibited higher chances of a moderate risk of bias than the others.

**Figure 6:**
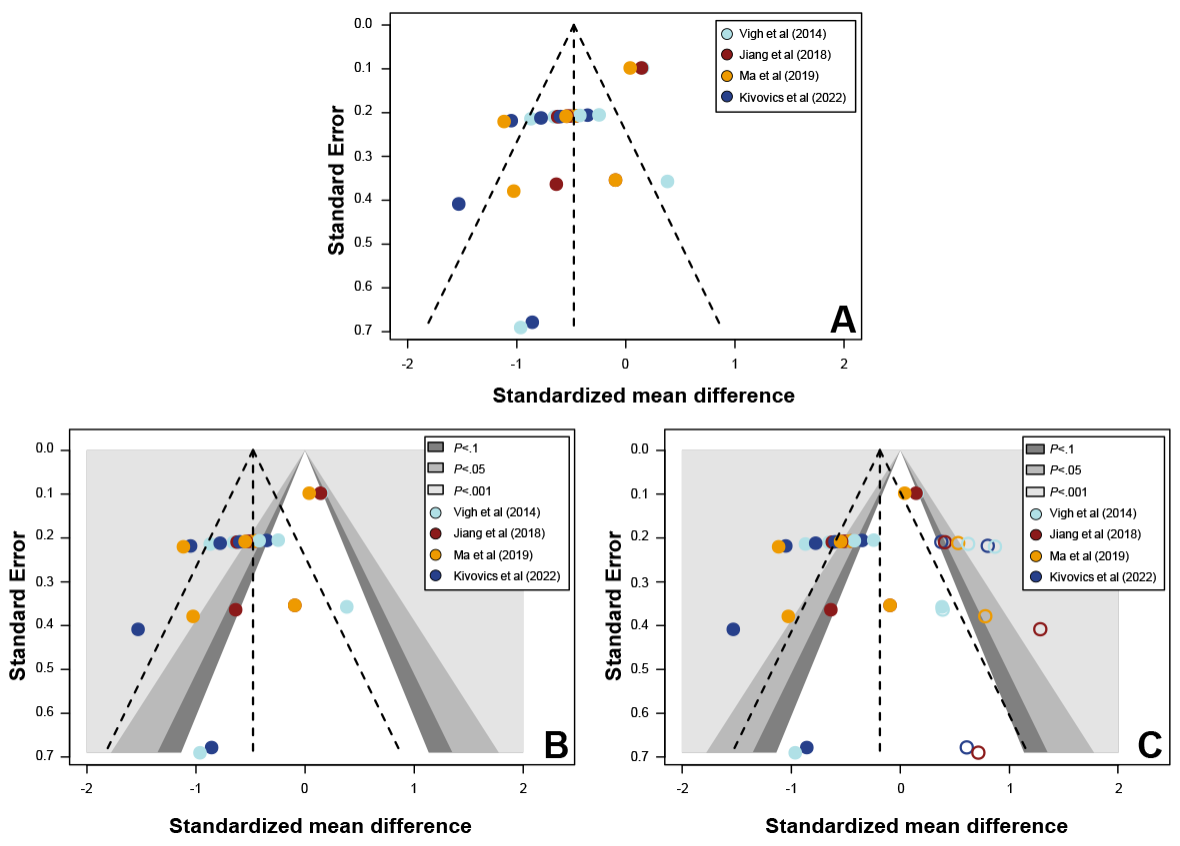
Funnel plots for publication bias assessment. (A) Funnel plot. (B) Contour-enhanced funnel plot. (C) Contour-enhanced funnel plot with the trim-and-fill method. Note: The dashed lines represent the random-effects estimate and the corresponding 95% confidence limits; the shaded regions represent different significance levels for the effect size. The filled circles indicate the observed data reported in the included studies, and the empty circles indicate the imputed and added data after Duval and Tweedie trim-and-fill analysis.

The funnel plot (Figure 6) and the result of Egger test indicated the presence of asymmetry (95% CI −4.78 to −1.93; *df*=−4.604; *P*<.001). Visual inspection of the contour-enhanced funnel plot was performed to assess potential publication bias. The asymmetry in the plots, which might be owing to the increased missing data present in the contours of the statistically significant region (gray shaded regions) than in the areas of nonstatistical significance (white region), suggests that the cause of the asymmetry might be because of factors other than publication bias such as variable study quality [48]. After adjusting for missing data using the Duval and Tweedie trim-and-fill method [34], 11 data points were added to adjust the asymmetry of the funnel plot and reduce the effects of existing publication bias (95% CI −2.89 to −.82; *df*=−1.097; *P*=.28).

### Positional Deviations of the Implant Placed With AR Navigation

**Figure 7:**
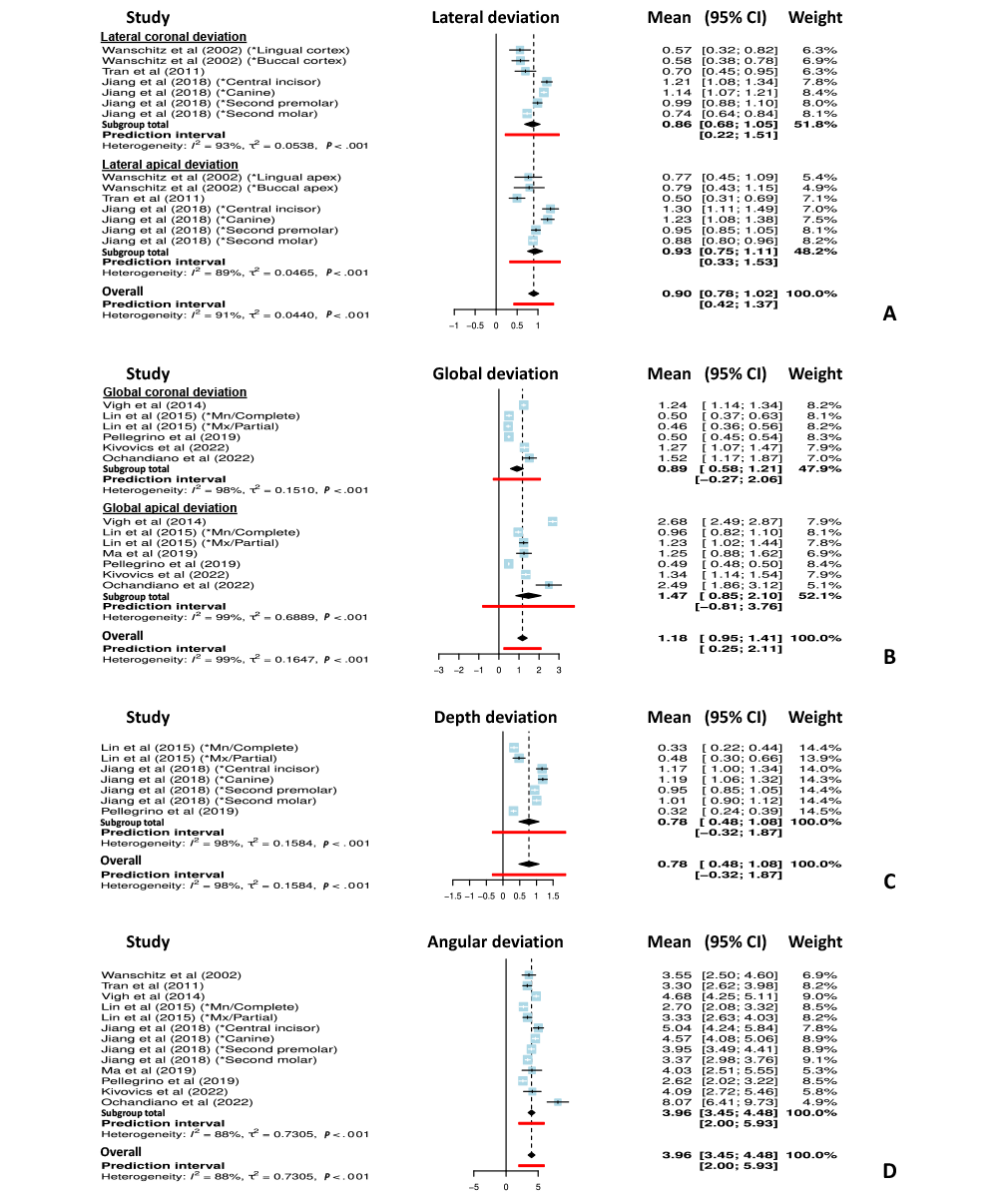
Forest plot showing the positional deviation of the implant placed using augmented reality navigation. (A) Lateral deviation (mm). (B) Global deviation (mm). (C) Depth deviation (mm). (D) Angular deviation (°). Mx=maxillary; Mn=mandibular.

A total of 346 implant sites were evaluated (181 implants, 59 drilling channels, and 106 parallel pins). Detailed descriptive characteristics of the AR-guided implant placement systems used in the included studies are presented in Table 3. The detailed positional deviations (mm) of the implants placed with AR navigation regarding the coronal, apical, and global deviations are shown in Figure 7. Accordingly, the pooled total weighted mean lateral deviation was 0.90 (95% CI 0.78-1.02) mm, with a deviation of 0.86 (95% CI 0.68-1.05) mm at the coronal region and 0.93 (95% CI.75-1.11) mm at the apex. The pooled total weighted global deviation was 1.18 (95% CI 0.95-1.41) mm, with a deviation of 0.89 (95% CI 0.58-1.21) mm in the coronal region and 1.47 (95% CI 0.85-2.10) mm at the apex. The pooled weighted depth deviations and angular deviations were 0.78 (95% CI 0.48-1.08) mm and 3.96° (95% CI 3.45°-4.48°), respectively.

### Comparations Between AR Navigation and the Other Implant Placement Methods

**Figure 8:**
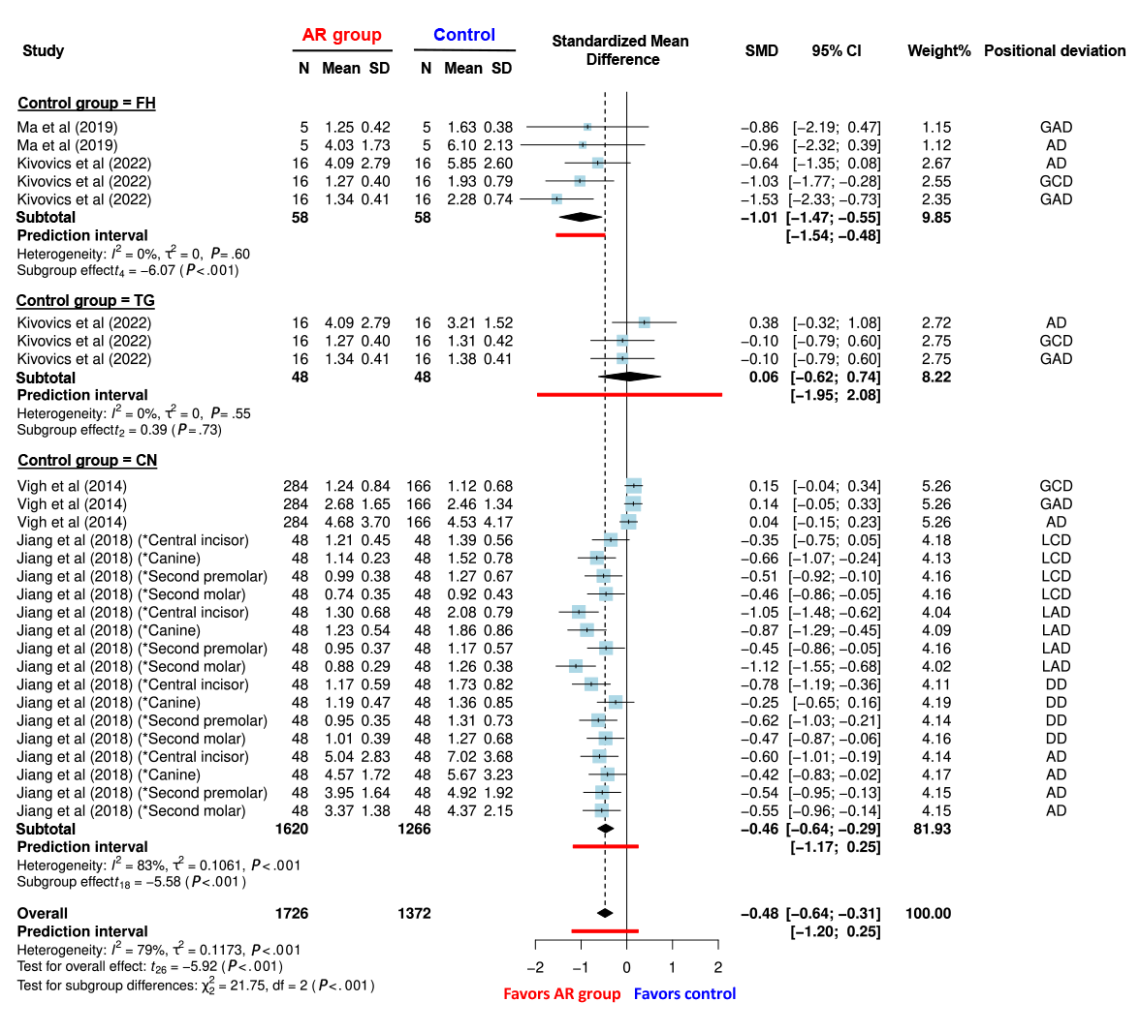
Forest plot comparing the positional deviation (mm) of implants placed using augmented reality (AR) navigation versus the freehand (FH), template-based static guidance (TG), and conventional navigation (CN) methods. AD: angular deviation; DD: depth deviation; GAD: global apical deviation; GCD: global coronal deviation; LAD: lateral apical deviation; LCD: lateral coronal deviation.

**Figure 9:**
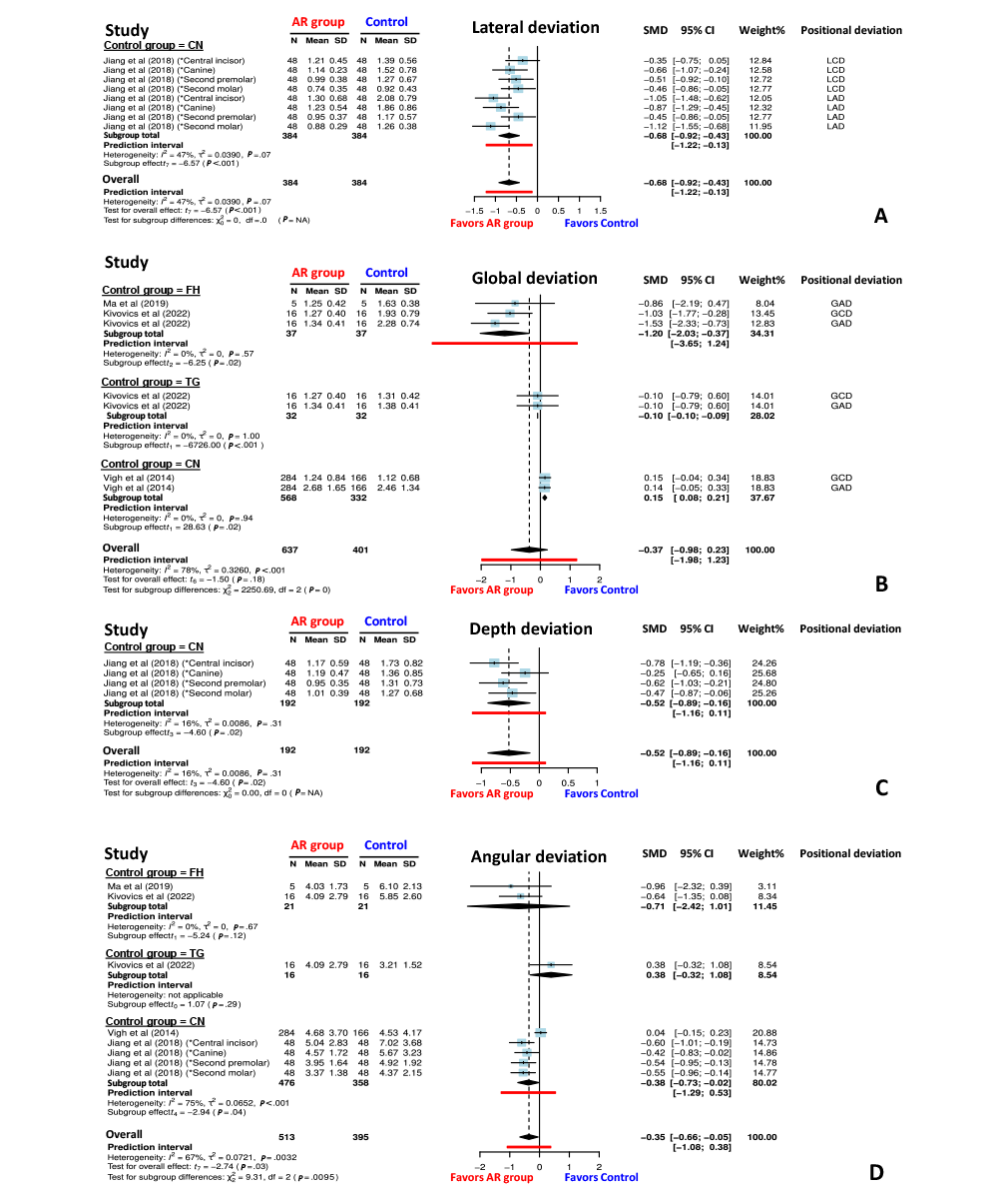
Forest plot comparing the positional deviations of the implants placed using augmented reality (AR) navigation versus the freehand (FH), template-based static guiding system (TG), and conventional navigation (CN) methods. (A) Lateral deviation (mm). (B) Global deviation (mm). (C) Depth deviation (mm). (D) Angular deviation (°). GAD: global apical deviation; GCD: global coronal deviation; LAD: lateral apical deviation; LCD: lateral coronal deviation.

Forest plots representing the SMD between the implant positional deviations in the AR navigation and control groups are shown in Figure 8. The AR navigation group showed significantly higher accuracy than the FH group (SMD=−1.01; 95% CI −1.47 to −0.55; *P*<.001) and CN groups (SMD=−0.46; 95% CI −0.64 to −0.29; *P*<.001), and similar accuracy to the TG group (SMD=0.06; 95% CI −0.62 to 0.74; *P*=.73). Detailed positional deviation comparisons are shown in Figure 9. For lateral deviation (mm), the AR navigation group exhibited significantly smaller deviations than the CN group (SMD=−0.68; 95% CI −0.92 to −0.43; *P*<.001; *I*^2^=47%). For global deviation (mm), the implants placed in the AR navigation group showed smaller deviations than those in the control group (SMD=−0.37; 95% CI −0.98 to 0.23; *P*=.18; *I*^2^=78%). The AR navigation group exhibited significantly smaller deviations than the FH group (SMD=−1.20; 95% CI −2.03 to −0.37; *P*=.02; *I*^2^=0%) and the TG group (SMD=−0.10; 95% CI −0.10 to −0.09; *P*<.001; *I*^2^=0%) but showed larger deviations than the CN group (SMD=0.15, 95% CI 0.08-0.21; *P*=.02; *I*^2^=0%). Regarding the depth deviations (mm), the AR navigation group exhibited smaller deviations than the CN group (SMD=−0.52; 95% CI −0.89 to −0.16; *P*=.02; *I*^2^=16%). Angular deviations (°) of the implants placed in the AR navigation group were also significantly smaller than those of the implants placed in the control groups (SMD=−0.35; 95% CI −0.66 to −0.05; *P*=.03; *I*^2^=67%). Specifically, the AR navigation group showed smaller deviations of placed implants than the CN group (SMD=−0.38; 95% CI −0.73 to −0.02; *P*=.04; *I*^2^=75%) and the FH group (SMD=−0.71; 95% CI −2.42 to 1.01; *P*=.12; *I*^2^=0%) but exhibited larger deviations than the TG group (SMD=0.38; 95% CI −0.32 to 1.08; *P*=.29).

## Discussion

### Accuracy of Implant Placed With AR Navigation

This systematic review aimed to evaluate the accuracy of AR navigation and compare it with that of FH, TG, and CN methods. The results of the meta-analysis indicated that the errors of implant placement using AR navigation were within the safety zone, which is recommended to be in the range of 1-2 mm horizontally and vertically [1,49-51] and up to an angle deviation of 5° [49]. The results also indicate a higher accuracy of AR navigation than the FH method. In addition, the accuracy of AR navigation was higher than that of the CN method, in which the operators needed to look away from the surgical sites to follow the guidance displayed on the computer screen. Although the implants placed using AR navigation exhibited global positional deviations similar to those of the implants placed using the TG method, they exhibited significantly larger angular deviations.

### Accuracy of AR Navigation in Comparison With Other Implant Placement Methods

The accuracy of TG implant surgery is superior to that of the FH method [52]. In addition, the accuracy of dynamic implant navigation systems is comparable with that of the static guiding method [10,53,54]. In this review, most of the introduced AR navigation methods were developed by integrating innovative AR technology into traditional implant navigation systems, in which AR was used to project the patient parameters, relevant radiographic images, 3D reconstruction of the preoperatively planned implant, or output of the navigation system screen to allow the surgeon to visualize real-time dynamic guidance without being forced to look away from the patient’s mouth [21,22,39,40,42,44,45]. The use of AR navigation provides surgeons with greater flexibility by allowing adjustments of the presurgical plan during real-time operation following the actual surgical site and oral conditions that would not have been possible with static guiding systems. In particular, in several advanced AR-assisted navigation systems, digital guidelines and warning alerts were included to prevent tolerances of the drills from the safety zone and planned implant positions [40,43-47]. Hence, it is expected to significantly improve the accuracy of traditional dynamic navigation. Moreover, AR technology can be combined with a surgical guide template to enhance both the accuracy and visualization of the implant guidance. Lin et al [46] reported a significant reduction in implant positional deviations by integrating surgical guide templates with AR technology.

### General Structure of an AR-Assisted Dental Navigation System

Similar to CN systems, AR navigation involves the same phase of preoperative implant planning based on CBCT and intraoral data of patients and requires a navigation system for tracking the real-time position of the surgical instruments. However, it uses innovative imaging technology in the form of AR to overlay the digital presurgical plan over the actual surgical site. Generally, the setting of an AR-assisted dental implant navigation system consists of 3 components: image registration, image tracking, and AR image display devices.

Marker-based and marker-free methods are the 2 main approaches used for image registration and tracking. For marker-based image matching, specific artificial markers that are attached to the reference template applied in the patient’s mouth and implant drilling instruments are used to merge the digital presurgical plan with the real operation site. In contrast, the marker-free approach uses a point cloud–based registration method and tooth shape tracking methods to merge digital images in a real environment. In this review, most reported AR navigation systems relied on marker-based image registration and image tracking techniques [20-22,39-46], and only 3 studies have reported marker-free AR navigation systems [23,41,47]. Compared with the marker-based registration method, marker-free methods do not require additional markers placed on the patient, which accelerates the registration speed, simplifies the surgical guiding process, reduces patient discomfort, and increases the surgeon’s convenience [47]. However, it is more challenging to use teeth as natural landmarks for tracking because they have less texture, are difficult to distinguish from the background noise using either structured light or stereo vision, occupy only a small portion of the camera view, and are easily covered by lips or other anatomical structures during the movement of the patient and surgical instrument [23,38].

As for AR image display, see-through HMD devices are the main tools [20,22,37,39,40,42,44-46]. Based on image display principles, HMD devices can be classified into optical see-through (OST) and video see-through (VST) HMD [55]. For OST HMDs, half-transparent mirrors placed in front of the user’s eyes are used to optically combine real and digital world images in the user’s eyes. HoloLens (Microsoft Corp) is a popular commercialized OST HMD that enables physicians to obtain immediate insight into patient information by overlaying it with the view of the clinical scenario. It helps medical students gain a better understanding of complex human anatomies or a better experience with treatment procedures and assists patients during rehabilitation and treatment [56]. In this review context, effective and successful applications of HoloLens as an image display component of an AR-assisted implant navigation system have been introduced in 2 studies [22,45]. In contrast to the OST HMD, VST HDMs use 2 miniature video cameras mounted on the headgear to capture real-world images and electronically combine them with digital images. In addition, some AR navigation systems allow AR images to be viewed through IV overlay devices with the surgeon’s naked eye [21,23,38,43,47]. The IV overlay display is a new tool for autostereoscopic display that uses a fast image rendering algorithm to project a digital image through a microconvex lens array using multiple rays so that the observer can view animated 3D objects from various directions as if they are fixed in 3D space [57]. In particular, a smartphone-based AR method for intraoperative implant visualization and final verification of implant position via a dedicated smartphone app was introduced in a study that relied on a marker-based tracking method [41].

### Limitations

This systematic review has some limitations, including the relatively small number of included studies owing to the limited research in this new area and some sources of bias. Another limitation is the risk of high heterogeneity among the included studies. The inconsistency in the included studies was owing to the uniformity of implant placement error measurements, as multiple variables (lateral coronal and apical, global coronal and apical, depth, and angular deviations) were considered. The variety in the selection of the control group (FH, TG, and CN groups) and the subjects of the experiment (human, cadaver, artificial model, and patient’s cast) also contributed to the high heterogeneity among the studies. Therefore, in this review, a random effects model was used to perform a meta-analysis and subgroup analyses were performed to reduce heterogeneity. Further studies that consider patient- and surgeon-related factors, such as user satisfaction, convenience, and comfort, should be conducted to further our understanding of the existing AR-assisted implant navigation systems.

### Conclusions

This systematic review supports the relevance of AR-assisted implant navigation methods. The meta-analysis showed that the accuracy of AR implant navigation was comparable with that of the highly recommended dental implant–guided surgery method, TG, and even superior to that of the conventional FH and CN methods. It should be noted that although AR implant navigation may be considered an effective immersive surgical guidance for dental implant placement, limited studies regarding the clinical application are available. Further studies that consider patient- and surgeon-related factors, such as user satisfaction, convenience, and comfort, should be conducted to further our understanding of the existing AR-assisted implant navigation systems.

## Appendix

Multimedia Appendix 1 Characteristics of the included studies.

## Abbreviations

AR: augmented reality
CAIS: computer-aided implant surgery
CBCT: cone-beam computer tomography
CN: conventional navigation
FH: freehand
GAD: global apical deviation
GCD: global coronal deviation
HMD: head-mounted display
IV: integral videography
LAD: lateral apical deviation
LCD: lateral coronal deviation
OST: optical see-through
PRISMA: Preferred Reporting Items for Systematic Reviews and Meta-Analyses
SMD: standardized mean difference
TG: template-based static guiding
VST: video see-through
