# Supplementary material for: Accuracy of Augmented Reality–Assisted Navigation in Dental Implant Surgery: Systematic Review and Meta-analysis
Source: J Med Internet Res. 2023 Jan 4;25:e42040. doi: 10.2196/42040 (PMC9856431; doi:10.2196/42040)
Supplement: Multimedia Appendix 1 [file jmir_v25i1e42040_app1.docx]

**Multimedia Appendix 1.** Characteristics of the included studies.

| Study | Jaws (n)/Edentulism | Implants or drill channels (n) | Overlaid virtual image | Tracking technology | Image display technology | Control group | Major findings |
| --- | --- | --- | --- | --- | --- | --- | --- |
| Wanschitz et al [39] | Mn (n=3)/Complete | 15 | Implant and the axis of the drill | Marker-based | HMD/OST | NR | The use of HMD allowed an average precision within 1 mm (range up to 3 mm) for the implant position and within 3° deviation for the implant inclination (range up to 10°). Control during the drilling procedure was significantly improved by stereoscopic vision through the HMD. |
| Yamaguchi et al [42] | Mn (n=1)/partial | 4 | The virtual preoperative simulation image of a dental implant placement position was overlaid onto the real surgical stent | Marker-based | Retinal projection HMD/Projector-based | NR | The developed procedure could be performed as a real time image overlay. With a view to the realization of the clinical applications, the size of markers, image overlay accuracy, measurable range, and light conditions should be evaluated in future studies. |
| Tran et al [43] | Mn (Phantom n=1; volunteer n=1)/Partial | 10 | Surgical path and nerve channel (Warning alert included) | Marker-based | IV overlay device/OST | NR | Instead of being displayed on a separate screen, 3D virtual presentations of osseous structures and soft tissues were projected onto the patient’s body, providing surgeons with exact knowledge of depth information of high-risk tissues inside the bone. Operational tasks performed using the study system showed an overall positional error of <1 mm. |
| Yamaguchi et al [20] | Mn (n=1)/partial | NR | Implant and the mandibular canal | Marker-based | Retinal projection HMD/Projector-based | NR | Development of a dental implant surgical navigation system that combines AR technology and the RID and its real time image overlay. By implementing the least median square method, a more robust calibration was achieved. Further development of the markers will be planned to decrease the reprojection errors using the other markers including known sampling points fixed on the patient’s oral cavity. |
| Vigh et al [44] | Mn (n=3)/complete | 10 virtual implants/450 drilling holes | Positions of pilot boreholes for dental implants were placed (Warning alert included) | Marker-based | HMD/VST | CN | The HMD had no major drawbacks compared with the monitor setting. The striking advantage was that the surgeon was no longer obliged to turn his head away from the operation site during navigation as all data relevant to the procedure were superimposed on the image of the real world in his field of view. Personal skills seemed to be crucial as the results showed remarkable differences among the test persons. |
| Katić et al [40] | Mn (n=1)/NR | 2 | The nerve and the drill axis in comparison with the intended implant planning data (virtual guidelines and warning alert included). | Marker-based | HMD/OST | CN | The system made the surgery easier and showed ergonomic benefits, as assessed by a questionnaire. All relevant phases were recognized reliably. The new calibration showed significant improvements, while the deviation of the realized implants was <2.5 mm. |
| Lin et al [46] | Mx (n=4)/partial;Mn (n=4)/Complete | 24 (Mn); 16 (Mx) | The planned implants and adjacent anatomical structures of the virtual 3D jawbone (virtual guidelines and warning alert included) | Marker-based | HMD/VST | NR | Deviation of implant placement from the planned position was significantly reduced by integrating surgical templates and AR technology. There was a statistically significant difference in apical deviation between the maxilla and mandible in this surgical simulation (*P*<.05). |
| Jiang et al [47] | Mn (n=12)/Complete | 96 | Planned surgical pathway, virtual drill, nerve channel (virtual guidelines and warning alert included) | Marker-free (point cloud-based registration) | IV overlay device/OST | CN | The implant showed <1.5 mm mean linear deviation and <5.5° angular deviation. AR-guided implantation showed smaller horizontal, vertical, and angular errors in the apical areas of the central incisor and the canine region. The surgery time using the AR-guided navigation method was significantly shorter than that using the 2D image-guided navigation method (*P*<.05). The volunteer experiment demonstrated that the preoperative 3D models in situ accurately overlaid onto the surgical site. |
| Matsuo et al [37] | Mx (n=1)/partial;Mn (n=1)/partial | 7 | The lingual aspect of the operating field was visualized using endoscopic imaging. | NR | HMD/OST | NR | During operation, the surgeon was able to see both direct view and integrated virtual images freely by changing the direction of their gaze. Using the HMD, the surgeon could gauge the depths for drilling both the buccal and lingual sides simultaneously. It was easy to confirm the preoperative simulations without looking back at the monitor. |
| Ma et al [21] | Mn (Model, n=5; volunteer, n=1)/Partial | 10 | Planned surgical pathway, virtual drill, nerve channel | Marker-based | IV overlay device/OST | FH | The developed AR navigation system had acceptable CBCT-patient registration and implant accuracy. The proposed method is expected to be applied in clinics. |
| Pellegrino et al [22] | Mx (n=2)/partial | 2 | Mirroring the dynamic navigation system screen. | Marker-based | HMD/holographic | NR | AR could be useful in dental implantology for displaying dynamic navigation systems.  this technology did not seem to noticeably affect the accuracy of the procedure, specific software applications should further optimize the results. |
| Wang, Shen, and Yang [38] | Mx (n=1)/partial;Mn (n=1)/partial | 1 | The surgical planning results including the implant’s position and orientation | Marker-free (teeth shape tracking) | IV overlay device/VST | NR | The target registration error of the overlay yielded an average error of <0.50 mm with a time cost of <0.5 s. A volunteer trial was also conducted to show the clinical feasibility. The proposed registration method does not rely on any external fiducial markers attached to the patient. It performs automatically to maintain a correct AR scene, overcoming the misalignment difficulty caused by the patient’s movement. |
| Shrestha et al [23] | Mx, Mn (n=40)/NR | NR | NR | Marker-free | IV overlay device/VST | NR | The proposed system improved the accuracy of convergence and processing time compared with the globally optimal ICP algorithm. The RANSAC algorithm was also employed to detect and remove the outlier on the estimation and reduce the influence of extreme points. |
| Kivovics et al [45] | Mn (n=12)/Complete | 48 | The planned implant position and three separate dots represented the entry point, the angle, and the depth of the drill (the three dots play as a guiding and warning alert system) | Marker-based | HMD/holographic | FH; TG | The implant positioning accuracy of AR-based dynamic navigation was comparable with that of static CAIS and superior to that obtained by the free-hand approach. |
| Ochandiano et al [41] | Mx, Mn (n=11)/Partial | 56 | The virtual planned implant overlaid on the patient’s anatomy. | Marker-based | Smartphone/OST | TG; CN | Smartphone-based AR visualization is a valuable tool for intraoperative visualization and final verification, although it is a difficult technique for guiding surgery. |

*Note:2D=two-dimensional; 3D=three-dimensional; AR=augmented reality; CAIS=computer-aided implant surgery; CBCT=cone-beam computed tomography; CN=conventional navigation system; FH=free-hand implant-guiding system; HMD=head-mounted display; ICP=iterative closest point; IV=integral videography; Mn=mandibular; Mx=maxillary; NR=not reported information; OST=optical see-through; random sampling consensus algorithm=random sampling consensus algorithm; RID=retinal image display; TG=template-based static guiding system; VST=video see-through.
